# Pharmacokinetics and Pharmacodynamics Evaluation of Amoxicillin Against *Staphylococcus pseudintermedius* in Dogs

**DOI:** 10.3390/pathogens13121121

**Published:** 2024-12-19

**Authors:** Ji-Soo Jeong, Jeong-Won Kim, Jin-Hwa Kim, Chang-Yeop Kim, Eun-Hye Chung, So-Young Boo, Su-Ha Lee, Je-Won Ko, Tae-Won Kim

**Affiliations:** 1 College of Veterinary Medicine (BK21 FOUR Program), Chungnam National University, 99 Daehak-ro, Daejeon 34131, Republic of Korea; jisooj9543@gmail.com (J.-S.J.); lilflflb@gmail.com (J.-W.K.); jinhwa926@g.cnu.ac.kr (J.-H.K.); 963ckdduq@gmail.com (C.-Y.K.); ksissb1293@gmail.com (E.-H.C.); labong1966@gmail.com (S.-Y.B.); suhai2729@gmail.com (S.-H.L.); 2 Division of Radiation Biomedical Research, Korea Institute of Radiological and Medical Science, 75 Nowon-ro, Nowon-gu, Seoul 01812, Republic of Korea; 3 Inhalation Toxicology, Jeongeup Campus, KIT, Jeongeup-si 580-185, Republic of Korea

**Keywords:** antimicrobial, PK/PD modeling, dosing, drug exposure, antibiotic resistance

## Abstract

Antibiotic resistance in bacteria from companion animals poses significant public health risks. Prudent antibiotic use, particularly through pharmacokinetics/pharmacodynamics modeling, is crucial for minimizing resistance. We investigated the pharmacokinetics/pharmacodynamics of amoxicillin (AMX) against *Staphylococcus pseudintermedius*. A pharmacokinetic study was conducted on healthy dogs subcutaneously injected with a dose of 15 mg/kg AMX. The antibacterial efficacy of AMX was evaluated against a standard strain from animals (KCTC 3344) and clinical isolates from dogs (B-2, B-7, and B-8), with minimum inhibitory concentrations (MICs) of 0.25, 0.5, 64, and 16 μg/mL, respectively. The half-life of AMX was 7 h, allowing for extended drug efficacy. The time above MIC (%T > MIC) values indicated that the AMX concentrations were maintained above MICs of the two susceptible strains (KCTC 3344 and B-2) for more than 80% of the time when dosed at a one-day interval, suggesting an effective treatment. The area under the curve over 24 h/MIC ratios confirmed the bacteriostatic, bactericidal, and bacterial eradication effects of AMX against *S. pseudintermedius* strains, except for B-7 (the most resistant strain). These results support improved clinical dosing strategies for AMX against *S. pseudintermedius* infections in dogs.

## 1. Introduction

Antibiotic resistance has become a major concern in veterinary medicine [1]. Particularly, antibiotic resistance in bacterial pathogens derived from companion animals poses a significant public health risk [2]. To improve the success of antibiotic therapy, appropriate dosing regimens that minimize adverse effects and reduce the emergence of resistance are essential [3]. This requires research into mechanisms involved in the changes in antibiotic concentrations (pharmacokinetics) and effects of antibiotics (pharmacodynamics).

Antibiotic resistance of *Staphylococcus* species in companion animals is increasing worldwide [4]. Specifically, *Staphylococcus pseudintermedius* is a coagulase-positive *Staphylococcus* species associated with dermatitis and otitis externa in dogs, and it acts as an opportunistic pathogen in many veterinary infections [5]. Staphylococcal dermatitis infections in companion animals often require prolonged and repeated antibiotic treatments [6]. Because treatment duration is a major factor that contributes to the development of resistance, the use of optimized antibiotic regimens that can reliably control infection is necessary to minimize resistance occurrence [7].

Previous studies have reported that antimicrobials such as amoxicillin (AMX) and cephalexin are effective against *S. pseudintermedius* isolates [8]. AMX is a time-dependent beta-lactam antibiotic used to treat bacterial infections in pets, including skin, respiratory, ear, and gastrointestinal infections [9]. The AMX dosing regimen varies depending on the type of animal and bacterial strain. Typically, AMX is administered as an oral tablet to treat *Staphylococcus* infections in dogs, and several studies have evaluated the pharmacokinetic properties of oral AMX in dogs [10,11]. However, to the best of the authors’ knowledge, few pharmacokinetic studies have analyzed injectable AMX in dogs, especially with long-acting formulations. In this study, a pharmacokinetic/pharmacodynamic (PK/PD) approach was demonstrated to evaluate the concentration–response relationship of AMX against the standard strain and three clinical isolates from dogs with different susceptibilities to AMX. The aim of this study was to assess the PK/PD parameters and efficacy of AMX against four *S. pseudintermedius* strains in dogs and propose optimal AMX dosing regimens for each strain, thereby verifying the practical differences in dosage on the basis of variations in strain susceptibility.

## 2. Materials and Methods

### 2.1. Chemicals and Reagents

Amoxicillin trihydrate standard was purchased from Sigma-Aldrich (St. Louis, MO, USA). Muller–Hinton broth (MHB), sodium chloride, and agar for bacterial cultures were also obtained from Sigma-Aldrich. AMX injection, a long-acting formulation manufactured by Samyang Anypharm Co. (Seoul, Republic of Korea), was used for the pharmacokinetic study. All the chemicals and solvents used in the experiments were of analytical grade with minimum 95% purity.

### 2.2. Bacterial Strains and Culture Conditions

The standard strain was obtained from the Korean Collection for Type Cultures (KCTC; Daejeon, Republic of Korea; KCTC No. 3344), and the *S. pseudintermedius* isolates (B-2, B-7, and B-8) were obtained from dogs at the Chungnam National University Animal Hospital (Daejeon, Republic of Korea). B-2 and B-7 were isolated from ear skin, whereas B-8 was isolated from urine. Detailed information on *S. pseudintermedius* clinical isolates from dogs is provided in Table S1. All the strains were stored in liquid nitrogen until further use. The strains were cultured in MHB and Muller–Hinton broth/agar (MHA) plates and incubated at 37 °C. Antibiotic resistance was assessed using the disk diffusion method [12].

### 2.3. Minimum Inhibitory Concentration (MIC)

MICs were evaluated using broth macrodilution and microdilution methods, according to the Clinical and Laboratory Standards Institute Guidelines [13]. *S. pseudintermedius* strains were cultured in 10 mL of MHB medium at 37 °C in a shaking incubator for 4 h. The absorbance was measured at 600 nm by using an ultraviolet–visible spectrophotometer (X-ma 1000; Human Co., Seoul, Republic of Korea) and diluted to 1 × 10^6^ CFU/mL by adjusting the absorbance to match the McFarland standard curve. Then, the diluted strains were mixed with the AMX-containing medium at a 1:1 ratio. The AMX stock solution was prepared in MHB medium at a concentration of 5120 μg/mL. Based on the resistance of each strain, a range of AMX test concentrations from 0.03125 to 512 μg/mL was used, achieved through a 2-fold serial dilution. The macrodilution method was conducted in sterile tubes with a final volume of 2 mL, whereas the microdilution method was performed in sterilized 96-well plates with a final volume of 200 μL. The AMX and bacterial mixtures were incubated at 37 °C for 24 h. The concentration in the test tube in which no turbidity was visually observed was determined as the MIC.

### 2.4. Minimum Bactericidal Concentration (MBC)

After determining the MIC of AMX, the MBC was determined by referring to the method described earlier [14]. For this, 10 μL of the *S. pseudintermedius* strain cultures at a concentration above the MIC was spread onto MHA plates and incubated at 37 °C for 24 h. The lowest concentration at which the number of colonies was reduced by 99.9% from the initial inoculum concentration of 5 × 10^5^ CFU/mL was determined to be the MBC. All tests were repeated thrice.

### 2.5. Mutant Prevention Concentration (MPC)

For MPC evaluation, as previously described [15], 4–5 colonies of *S. pseudintermedius* strains of equal size were selected and incubated in MHB at 37 °C overnight. The absorbance at 600 nm was plotted on a McFarland standard curve to determine the bacterial count, and the culture was diluted to a concentration of 1 × 10^10^ CFU/mL. A 200 μL aliquot of the diluted culture was spread on MHA plates containing AMX (0.125–1024 μg/mL) and incubated at 37 °C for 72 h. The minimum concentration at which no colonies were observed was determined as the MPC. All tests were repeated thrice.

### 2.6. Post-Antibiotic Effect (PAE)

To determine the duration of PAE, the standard method of Craig and Gudmundsson was used [16]. Briefly, standard strain KCTC 3344 was incubated in MHB medium at 37 °C for 2 h. Then, 1 × 10^6^ CFU/mL of bacteria was mixed with AMX at a 1:1 ratio, with concentrations corresponding to 1×, 2×, and 4× MIC, and incubated at 37 °C for 1 h. After 1 h of exposure, the mixture was centrifuged, and 10 μL of the pellet was diluted 1:1000 in MHB, spread on MHA plates, and colony counts were determined at 0, 1, 2, 4, 6, 8, 10, and 12 h. All tests were performed in triplicate.

### 2.7. Animals and Experimental Design

A pharmacokinetic study of AMX in dogs was conducted at KULF Inc. (Namyangju, Republic of Korea; KULF-IACUC-2022-04). Five healthy male beagles (10 months of age, 8–10 kg) were housed indoors in a climate-controlled facility, in accordance with the accepted guidelines for the care and use of laboratory animals. The test substance was a long-acting AMX formulation (amoxicillin trihydrate, 150 mg/mL) and was administered as a single subcutaneous injection at a concentration of 15 mg/kg according to established dosing regimen guidelines for the long-acting formulation of AMX for dogs [17]. Blood samples were collected in K_2_-ethylenediaminetetraacetic acid tubes at 0, 0.25, 0.5, 1, 2, 4, 6, 8, 10, 12, 24, and 30 h to determine the plasma concentration of AMX. The collected blood was centrifuged to obtain plasma, which was stored at −80 °C until analysis.

### 2.8. Plasma Sample Extraction Procedure

AMX stock and working solutions were dissolved in distilled water (DW). Acetonitrile (ACN; 300 μL) was added to 38 μL of dog plasma, vortexed for 10 min, and centrifuged at 15,520× *g* for 10 min at 4 °C. Then, 280 μL of the supernatant was concentrated under nitrogen at 40 °C and suspended in 50 μL of DW. The final extract was passed through a 0.20 μm polyvinylidene fluoride membrane filter and donated for analysis.

### 2.9. Ultra-Performance Liquid Chromatography–Tandem Mass Spectrometry (UPLC-MS/MS) Analysis

AMX was detected and quantified using UPLC-MS/MS (Agilent Technologies, Santa Clara, CA, USA). Chromatographic separation was performed on an Eclipse Plus C18 2.1 × 100 mm, 3.5 μm column (Agilent Technologies) at a column temperature of 40 °C. The mobile phase consisted of 0.1% formic acid in DW (solvent A) and 0.1% formic acid in ACN (solvent B) at a flow rate of 0.3 mL/min, with the following gradient elution: 0 min, 10% solvent B; 0−3 min, 10−90% solvent B; 3−4 min, 90−10% solvent B. Multiple reaction monitoring with *m*/*z* transitions of 366.1 → 134 and 113.8 at collision energy 40 and 44 eV, respectively, was used in positive ionization mode. The ion source temperature was set to 250 °C, and the nitrogen gas flow rate was set at 11 mL/min. The sample injection volume was 5 μL.

A calibration curve was generated using various standard concentrations prepared by spiking blank dog plasma samples. The method for the analysis of AMX concentration in dog plasma was validated according to the “Guideline on Bioanalytical Method Validation” of the Korean Ministry of Food and Drug Safety [18]. Method validation evaluated the calibration curve, specificity, matrix effect, accuracy, precision, and stability and defined signal-to-noise (S/N) ratios of 3 and 10 as the limit of detection (LOD) and limit of quantitation (LOQ), respectively. Quality control (QC) samples were also prepared at concentrations of 100, 500, and 1000 ng/mL by using the same method as the calibration standard, and intra- and inter-day recoveries were calculated at these three concentrations to evaluate the accuracy and precision.

### 2.10. Pharmacokinetic Data Analysis

The pharmacokinetic parameters were calculated on the basis of a non-compartmental model by using Monolix 2023R1 (Lixoft SAS, Simulations Plus Inc., Lancaster, CA, USA). The maximum concentration (C_max_) and time to reach C_max_ (T_max_) were obtained directly from the individual plasma concentration-versus-time profiles. The elimination half-life (T_1/2_) was obtained by linear regression of the log-transformed concentration data. The area under the curve from time zero to the last sampling time (AUC_0−t_) was estimated using the linear log-trapezoidal and linear up-log-down rules. Data for T_max_ are presented as median and range values. Other data are presented as mean ± standard deviation values.

### 2.11. Ex Vivo Time-Killing Curve Assay

The time-kill assay was performed as previously reported [19], where four to five colonies of similarly sized *S. pseudintermedius* strains were selected and incubated in MHB at 37 °C for 4 h in a shaking incubator. The turbidity of the culture was measured at 600 nm and diluted to a concentration of 1 × 10^6^ CFU/mL. The diluted bacteria were mixed at a 1:1 ratio with dog plasma from each time point obtained from the AMX pharmacokinetic experiments and incubated at 37 °C. A 10 μL sample of each culture was collected at 0, 1, 2, 4, 8, 12, and 24 h, diluted with MHB to a concentration of 10^1^−10^7^ CFU/mL, and incubated dropwise on MHA plates at 37 °C for 16−24 h. The LOD was 10 CFU/mL, and the results were plotted as CFU/mL over time. Each test was repeated twice for each strain.

### 2.12. PK/PD Relationship of AMX Against S. pseudintermedius

The area under the curve over 24 h (AUC_24h_)/MIC ratio was used as a predictive PK/PD index [20,21], and the correlation between the antimicrobial activity and AUC_24h_/MIC ratio of AMX against *S. pseudintermedius* strains was determined using a sigmoid maximum effect (E_max_) model as follows:

$$\mathrm{Log}\;\mathrm{Reduction}=E_{0}+\frac{E_{\max}\;\left(\mathrm{AUC}/{\mathrm{MIC}}^{n}\right)}{{EC50}^{n}+(\mathrm{AUC}/{\mathrm{MIC}}^{n})}$$

*E*_0_, log_10_ change in bacterial count in drug-free control;
*EC*50, *AUC*_24h_/*MIC* ratio required to achieve 50% of the E_max_;
*n*, Hill coefficient representing the slope of dose–response curve.

The AUC_24h_/MIC ratios required for the bacteriostatic (E  =  0), bactericidal (E = −3), and bacterial eradication (E = −4) effects were calculated.

### 2.13. Protein Binding of AMX in Dog Plasma

The binding ratio of AMX to dog plasma protein was determined using UPLC-MS/MS with Nanosep^®^ Centrifugal Filters (Pall Corporation, Port Washington, NY, USA) [22]. The filter was washed with 5% tween 20 and phosphate-buffered saline (PBS), and AMX at the same concentration was added to filter tubes with blank dog plasma samples and PBS and incubated at 37 °C for 1 h. The mixture was then centrifuged. After analyzing AMX concentrations in dog plasma and PBS before and after filtration, the protein-binding ratio of AMX in dog plasma was calculated using the following equation:

$${ƒ}_{u}=\frac{C_{FP}}{(1-\frac{C_{\mathrm{TPBS}}-C_{\mathrm{FPBS}}}{C_{\mathrm{TPBS}}})\times C_{\mathrm{TP}}}$$

$$\%\;\mathrm{Protein}\;\mathrm{Binding}=100\times (1-{ƒ}_{u})$$

*ƒ_u_*, free fraction;
*C_TP_*, total plasma concentration; *C_FP_*, filtered plasma concentration;
*C_TPBS_*, total PBS concentration; *C_FPBS_*, filtered PBS concentration.

### 2.14. Dose Estimations

The optimal AMX regimen for each *S. pseudintermedius* strain was calculated for bacteriostatic (E = 0), bactericidal (E = −3), and bacterial eradication (E = −4) by using the following equation [23]:

$$\mathrm{Dose}=\frac{\left(\mathrm{AUC}/\mathrm{MIC}\right)\cdot {\mathrm{MIC}}_{90}\cdot \mathrm{Cl}\;}{{ƒ}_{u}\cdot F}$$

*AUC*, area under the curve;
*AUC/MIC*, targeted endpoint for optimal efficacy;
*MIC*_90_, MICs required to inhibit the growth of 90% of bacteria;
*Cl*, clearance; *ƒ_u_*, free fraction; *F*, bioavailability.

## 3. Results

### 3.1. In Vitro Susceptibility Test

The MIC, MBC, and MPC values of the four *S. pseudintermedius* strains are summarized in Table 1. In KCTC 3344, AMX showed the lowest MIC and MBC values, both 0.25 μg/mL, whereas B-7 exhibited the highest resistance to AMX, with MIC = 64 μg/mL and MBC = 128 μg/mL. Additionally, MPC was measured to evaluate the potential of suppressing resistance trends to AMX, and MPC = 4 μg/mL was observed in only KCTC 3344.

### 3.2. In Vitro Time-Killing Curves and PAE Determination

**Figure 1:**
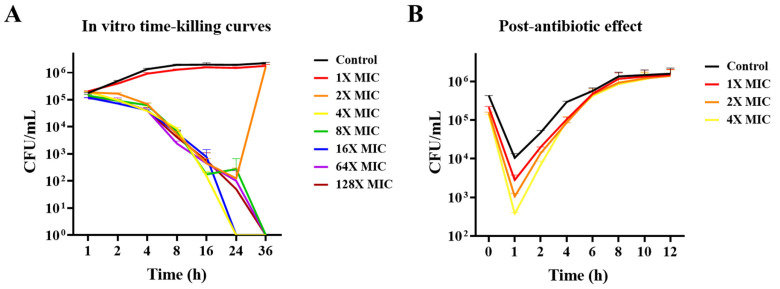
In vitro time-killing curves and post-antibiotic effect (PAE) values of amoxicillin (AMX) against standard strain, KCTC 3344. (**A**) In vitro time-killing curves were established after exposure to AMX for each time point at 1–128× MIC against KCTC 3344. (**B**) PAEs were measured after 1 h exposure to AMX at 1×, 2×, and 4× MIC against KCTC 3344. MIC, minimum inhibitory concentration.

When standard strain KCTC 3344 was treated with AMX at concentrations above 2× MIC (0.5 μg/mL), a rapid killing effect was observed (Figure 1A). However, at 2× MIC, bacterial regrowth occurred after 24 h, with bacterial density reaching levels similar to those in the control group. At a concentration of 4–128× MIC, a higher level of killing activity was maintained until 36 h.

Additionally, when the PAE of AMX against KCTC 3344 was measured (Figure 1B), PAE occurred after 1 h of exposure to 1×, 2×, and 4× MIC of AMX, followed by its removal, with a duration of approximately 1 h.

### 3.3. Pharmacokinetic Profiles of AMX in Dogs

According to UPLC-MS/MS, the LOD and LOQ of AMX in dog plasma were 9 and 28 ng/mL, respectively. The inter- and intra-day accuracy and precision of AMX in the QC samples at the three concentrations were 82.8–104.8% and 93.3–100.9%, respectively. The detailed validation results are presented in Table S2.

**Figure 2:**
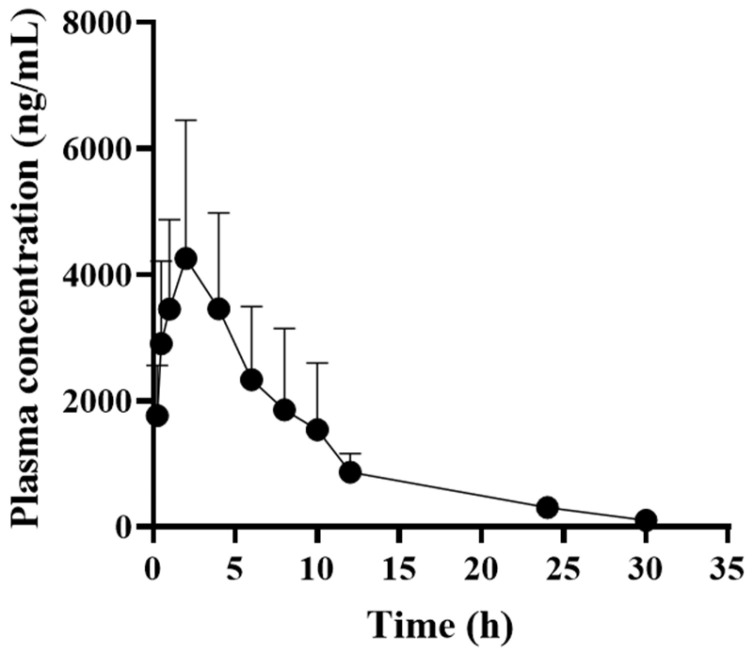
Mean plasma concentration vs. time profile of amoxicillin after subcutaneous injection of a single dose of 15 mg/kg to healthy dogs. Data expressed as mean ± standard deviation (*n* = 5) for each time point.

The time-dependent plasma concentrations of AMX in dogs after a single subcutaneous injection of a long-acting AMX formulation are shown in Figure 2, and the pharmacokinetic parameters are listed in Table 2. The C_max_ and AUC, representing the extent of systemic exposure, were 4309.3 ± 2161.1 ng/mL and 37,802.1 ± 14,719.8 h × ng/mL, respectively, with a half-life of approximately 7 h. The time above MIC (%T > MIC) values calculated for KCTC 3344 and B-2 were 110.2 ± 9.8% and 80.7 ± 6.5%, respectively, whereas the B-7 and B-8 values could not be determined.

### 3.4. Ex Vivo Antimicrobial Activity

**Figure 3:**
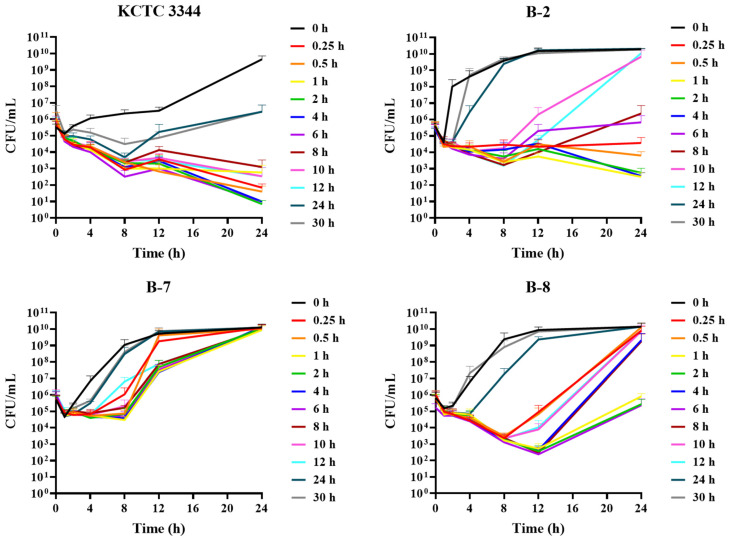
Ex vivo activity of amoxicillin for *Staphylococcus pseudintermedius* in plasma samples from the dogs injected subcutaneously with 15 mg/kg of AMX. KCTC 3344, standard strain; B-2, B-7, and B-8, clinical isolates from dogs.

To investigate the ex vivo antimicrobial activity of dog plasma against *S. pseudintermedius* strains, the standard strain KCTC 3344 and three isolates from dogs (B-2, B-7, and B-8) were used (Figure 3). In the case of KCTC 3344, which exhibited the lowest MIC (0.25 μg/mL), bacterial counts were below the LOD in plasma samples collected up to 12 h after 12 h of incubation. Regrowth was observed in only plasma samples collected at 24 and 30 h after 24 h of incubation. For B-2 (MIC 0.5 μg/mL) and B-8 (MIC 16 μg/mL), plasma samples collected 10 and 4 h after 24 h of incubation, respectively, showed levels of regrowth similar to the control group. Notably, B-7, which showed the highest resistance (MIC 64 μg/mL), exhibited the fastest bacterial regrowth in all plasma samples.

### 3.5. PK/PD Analysis

**Figure 4:**
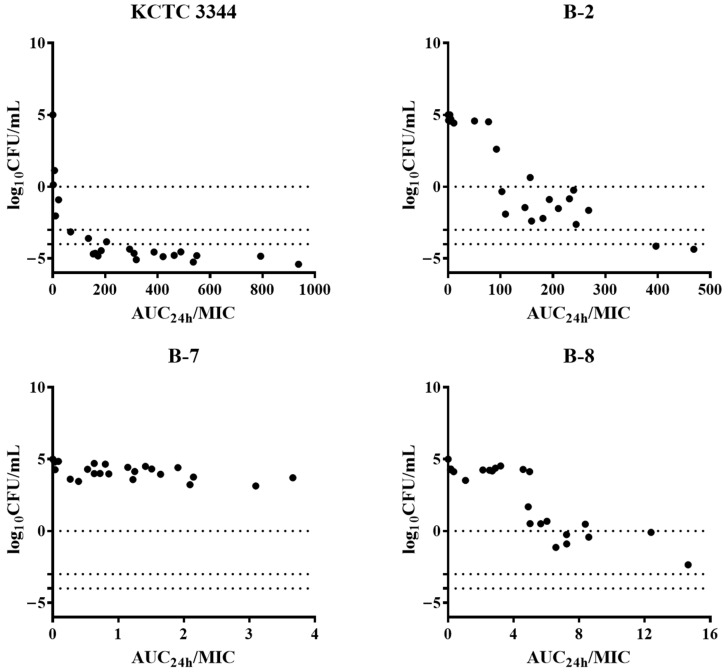
Pharmacokinetic/pharmacodynamic relationships of amoxicillin (AMX) with *Staphylococcus pseudintermedius*. Correlation plot of ex vivo antimicrobial activity and area under the curve over 24 h/minimum inhibitory concentration (AUC_24h_/MIC) ratio of AMX on the basis of the sigmoid maximum effect (E_max_) equation. The points represent observed values of dog plasma samples collected at time points from 0 to 30 h. KCTC 3344, standard strain; B-2, B-7, and B-8, clinical isolates from dogs.

The relationship between the PK/PD index, AUC_24h_/MIC ratio, and ex vivo activity was analyzed using the sigmoid E_max_ model. Based on this analysis, the simulated doses for each strain, adjusted for a 30% binding rate of AMX to dog plasma proteins, are presented in Figure 4 and Table 3. In the standard strain KCTC 3344, E_max_ was achieved with a bacterial reduction of 5.2 ± 0.3 log_10_ CFU/mL in dog plasma after subcutaneous administration of 15 mg/kg of AMX. The AUC_24h_/MIC ratios of AMX required to achieve bacteriostatic, bactericidal, and bacterial eradication effects in dog plasma for KCTC 3344 were 8.5 ± 4.6, 41.1 ± 23.2, and 104.0 ± 75.6 h, respectively. For the less susceptible clinical isolate B-2, the AUC_24h_/MIC ratios necessary to achieve bacteriostatic, bactericidal, and bacterial eradication effects were around 85.6 ± 11.9, 180.5 ± 8.8, and 307.9 ± 20.5 h, respectively, and these values were relatively higher than those for KCTC 3344. For B-8, only the bacteriostatic AUC_24h_/MIC ratio could be determined, and for B-7, which exhibited the highest resistance, the AUC_24h_/MIC ratio could not be determined.

## 4. Discussion

Antibiotic resistance of bacteria originating from companion animals is an emerging issue that increases public health risks [24], as the resistant bacteria can be transmitted between animals and humans through direct or indirect contact [25,26,27]. The prudent use of antibiotics to minimize resistance is becoming increasingly important, and research on the isolation of resistant pathogens from pets is gaining increasing attention [28]. In particular, PK/PD analyses of antibiotics against bacteria have been used to optimize antibiotic dosing regimens [29]. PK/PD modeling can help establish susceptibility breakpoints and predict optimal dosing schedules, such as dose and frequency. In this study, we conducted a PK/PD analysis of AMX against *S. pseudintermedius*, the bacterium most commonly isolated from skin lesions in pets. We investigated the antimicrobial activity of AMX against both the standard strain (KCTC 3344) and clinical isolates obtained from dogs (B-2, B-7, and B-8). On the basis of the derived PK/PD indices, we proposed an optimal dosing regimen for AMX.

Time-dependent antibiotics, such as AMX, are considered effective when the period during which the drug concentration remains above the MIC exceeds 40% of the dosing interval [30]. In the present study, the %T > MIC value showed that the duration was more than 80% when dosed at one-day intervals, confirming that the drug concentration remained above the MIC for the four *S. pseudintermedius* strains for more than 40% of the dosing interval, which is considered effective. Additionally, based on the 40%T > MIC threshold, the dosing interval for the KCTC 3344 (MIC 0.25 μg/mL) and B-2 (MIC 0.5 μg/mL) were calculated to be approximately 66 and 48 h, respectively. Previous studies have emphasized the importance of optimizing dosing strategies, such as shortening the dosing interval, to maintain antibacterial efficacy and prevent resistance development as bacterial susceptibility to AMX decreases, especially for time-dependent β-lactam agents [31]. Consistent with the previous reports, the results of the present experiment similarly demonstrated the necessity of adjusting the dosing interval based on bacterial susceptibility to AMX.

Beta-lactam antibiotics generally induce minimal or no PAE against Gram-negative bacteria, while typically causing a short PAE of less than 2 h against Gram-positive bacteria [32]. Similarly, in this study, the PAE of AMX against the KCTC 3344 strain was observed to be approximately 1 h. This is slightly shorter than the previously reported PAE of AMX with clavulanic acid against *S. aureus*, which was approximately 2 h [33]. In this regard, a short PAE, combined with the susceptibility of the target bacteria, may require frequent dosing to ensure the effective therapeutic efficacy of AMX. However, it has been reported that even antibiotics with a short PAE can maintain their effectiveness by prolonging the duration of bacterial growth inhibition through extended exposure times [34]. Typically, AMX is administered orally in tablet form, in combination with clavulanic acid [35]. In this study, we used a long-acting injectable formulation of AMX without clavulanic acid to address economic issues in drug development, and it was administered subcutaneously to dogs. The half-life identified in this study (7 h) was longer than that reported for oral administration to dogs (2 h), cats (1 h), and pigs (4 h) [36,37,38] and longer than that of koalas (4 h) [39]. This indicates that the drug remained in the body for a longer period after administration. In this current study, the long-acting formulation of AMX demonstrated an extended half-life, and it was shown that effective plasma concentrations of AMX can remain above the MIC for an extended period even after a single dose, which can help antibiotic effectiveness with relatively long duration.

In general, for beta-lactam antibiotics with a short half-life, maximizing exposure time can enhance efficacy, making %T > MIC the PK/PD index most closely associated with antibacterial activity [40]. However, Nielsen et al. [41] reported that depending on study design factors such as drug half-life and dosing interval, the AUC/MIC index may serve as a better predictor of drug efficacy when the %T > MIC parameter does not correlate well. In the present study, the AUC/MIC index was selected as the predictive PK/PD parameter in the ex vivo assay, which showed a better fit. According to the ex vivo antimicrobial activity results of this study, a complete bactericidal effect of AMX was observed against KCTC 3344 in dog plasma, whereas bacterial regrowth was noted in all three clinical isolates. Additionally, the bacteriostatic, bactericidal, and bacterial eradication effects of AMX were confirmed in KCTC 3344 and B-2 but not in the most resistant clinical isolate, B-7. On the basis of the derived AUC_24h_/MIC ratios, the optimal subcutaneous dose of AMX for KCTC 3344, B-2, and B-8 was calculated as described previously [23]. For KCTC 3344, the required doses for bacteriostatic, bactericidal, and bacterial eradication effects were approximately 1, 4, and 10 mg/kg, respectively, which are far less than the AMX single dose. For B-2, only the bacteriostatic dose, calculated to be approximately 16 mg/kg, was within the range of the currently used dosage, whereas the doses required for bactericidal and bacterial eradication effects were more than twice the amount. An MBC/MIC ratio of less than 4 indicates a bactericidal effect, whereas a ratio between 4 and 32 indicates a bacteriostatic effect [42]. The MBC/MIC ratio of AMX to B-2 was 8, which categorizes it as bacteriostatic. In veterinary medicine, the standard intravenous injection dose of AMX (20 mg/kg every 8 h) provides a pharmacokinetic/pharmacodynamic cutoff of 1 mg/L in healthy dogs and 4 mg/L in diseased dogs [11]. However, our results indicate that, for resistant strains, concentrations higher than the standard dosing regimen are necessary.

In clinical practice, bacterial strains with varying levels of resistance are commonly encountered. Similarly, in our results, the clinical isolates from dogs exhibited such high resistance to AMX that their MPC could not be determined. To effectively manage resistant clinical strains, it is necessary to consider combination therapy to overcome the limitations of single antibiotics. Additionally, exploring alternative antimicrobials or new treatment options is crucial. Notably, *S. pseudintermedius* is often found in conjunction with other pathogens in dog skin infections. Given the co-existence of susceptible and resistant bacterial strains, both must be carefully considered to select the optimal antibiotic regimen. This highlights the need for further veterinary clinical research to facilitate effective clinical application.

## 5. Conclusions

This study aimed to evaluate the PK/PD parameters and efficacy of AMX against *S. pseudintermedius* strains in dogs. It was confirmed that as the susceptibility of *S. pseudintermedius* strains decreases, maintaining antimicrobial efficacy requires either shortening the dosing interval or increasing the dosage of AMX. These findings provide a crucial foundation for establishing effective AMX dosage regimens for the treatment of *S. pseudintermedius* infections in dogs and designing more efficient clinical trials.

## Figures and Tables

**Table 1:** Minimum inhibitory concentrations (MICs), minimum bactericidal concentrations (MBCs), and mutant prevention concentrations (MPCs) of amoxicillin against *Staphylococcus pseudintermedius*. KCTC 3344, standard strain; B-2, B-7, and B-8, clinical isolates from dogs.

	KCTC 3344	B-2	B-7	B-8
MICs (μg/mL)	0.25	0.5	64	16
MBCs (μg/mL)	0.25	4	128	64
MPCs (μg/mL)	4	-	-	-

**Table 2:** Pharmacokinetic profiles of amoxicillin after subcutaneous injection of a single dose of 15 mg/kg to healthy dogs (*n* = 5).

Pharmacokinetic Parameters	
T_1/2_ (h)	7.1 ± 2.1
T_max_ (h) ^a^	2.0 (0.5 − 2.0)
C_max_ (ng/mL)	4309.3 ± 2161.1
AUC_last_ (h × ng/mL)	37,802.1 ± 14,719.8
AUC_INF_ (h × ng/mL)	39,782.0 ± 13,645.2
AUC_%Extrap (%)	6.1 ± 5.8
MRT_last_ (h)	7.6 ± 1.3
AUMC_last_ (h × h × ng/mL)	274,927.2 ± 74,853.4
Vd/F (mL/kg)	4558.7 ± 2726.6
Cl/F (mL/h/kg)	414.8 ± 144.0
K_el_ (1/h)	0.1 ± 0.0

AUC_last_, area under the curve from the time of dosing to the last measurable positive concentration; AUC_INF_, area under the curve extrapolated from the time of dosing to infinity; AUC_%Extrap, percentage of AUC_INF_ due to extrapolation from T_last_ to infinity; AUMC_last_, area under the moment curve from the time of dosing to the last measurable concentration; Cl/F, apparent clearance; C_max_, maximum plasma concentration; F, oral bioavailability; K_el_, elimination rate constant; MRT_last_, mean residence time from the time of dosing to the time of the last measurable concentration; T_1/2_, half-life; T_max_, time to peak concentration; Vd/F, apparent volume of distribution. ^a^ Data for T_max_ are presented as median (range); other data are presented as mean ± standard deviation.

**Table 3:** Pharmacokinetic/pharmacodynamic (PK/PD) analysis and area under the curve over 24 h/minimum inhibitory concentration (AUC_24h_/MIC) ratio values of amoxicillin (AMX) required to achieve bacteriostatic, bactericidal, and eradication effects against *Staphylococcus pseudintermedius* in dog plasma, and the predicted daily doses of AMX against *S. pseudintermedius* in dogs. KCTC 3344, standard strain; B-2 and B-8, clinical isolates from dogs.

PK/PD Parameters	KCTC 3344	B-2	B-8
E_max_ (log_10_ CFU/mL)	−5.2 ± 0.3	−4.6 ± 0.2	−1.3 ± 1.1
EC_50_ (log_10_ CFU/mL)	10.3 ± 7.1	85.3 ± 9.6	4.6 ± 2.6
N	1.0 ± 0.5	2.1 ± 0.4	6.6 ± 2.5
AUC_24h_/MIC for bacteriostatic (h)	8.5 ± 4.6	85.6 ± 11.9	6.6 ± 1.3
AUC_24h_/MIC for bactericidal (h)	41.1 ± 23.2	180.5 ± 8.8	-
AUC_24h_/MIC for eradication (h)	104.0 ± 75.6	307.9 ± 20.5	-
**Predicted doses (mg/kg·bw)**	**KCTC 3344**	**B-2**	**B-8**
Bacteriostatic (E = 0)	0.97 ± 0.54	16.31 ± 2.60	41.04 ± 9.00
Bactericidal (E = −3)	4.40 ± 2.21	34.77 ± 4.98	-
Eradication (E = −4)	10.22 ± 5.82	59.64 ± 9.38	-

EC_50_, AUC_24h_/MIC ratio required to achieve 50% of the E_max_; E_max_, maximal activity; N, Hill coefficient representing the slope of dose–response curve. Bacteriostatic, bactericidal, and eradication were defined as the net static, 3-log10, and 4-log10 kill effects after 24 h of incubation.

## Data Availability

The datasets generated and analyzed in the current study are available from the corresponding authors on reasonable request.

## Supplementary Materials

The following supporting information can be downloaded at https://www.mdpi.com/article/10.3390/pathogens13121121/s1, Table S1: Information about clinical isolates of *Staphylococcus pseudintermedius* isolated from dogs; Table S2: Validation results of amoxicillin (AMX) analysis method by accuracy and precision of each intra-day and inter-day analysis.
