# Supplementary material for: Pharmacokinetics and Pharmacodynamics Evaluation of Amoxicillin Against Staphylococcus pseudintermedius in Dogs
Source: Pathogens. 2024 Dec 19;13(12):1121. doi: 10.3390/pathogens13121121 (PMC11679838; doi:10.3390/pathogens13121121)
Supplement: Supplementary file 1 [file pathogens-13-01121-s001.zip › pathogens-3292434-supplementary.pdf]

Supplementary Table S1. Information about clinical isolates of *Staphylococcus pseudintermedius* isolated from dogs.

Strain	No.	Breeds	Sex	Age	Disease	Date of visit	Source	Date of isolation	Bacterial isolation
B-2	CNUAH-23-SI-011	Labrador retriever	CM	11 year	Otitis externa	10/20/23	Ear skin	10/20/23	O
B-7	CNUAH-23-SI-016	Mix	M	12 year	Hyperadrenocorticism	11/16/23	Ear skin	11/23/23	O
B-8	CNUAH-23-SI-017	Jindo	SF	12 year	Hepatocellular carcinoma	11/30/23	urine	11/30/23	O

Supplementary Table S2. Validation results of amoxicillin (AMX) analysis method by accuracy and precision of each intra-day and inter-day analysis.

Analyte	QC (ng/mL)	Day	Recovery			Inter-day		Intra-day	
			1	2	3	Mean recovery (%)	CV (%)	Mean recovery (%)	CV (%)
AMX	100	1	104.1	104.8	105.5	104.8	0.7	93.3	10.8
		2	95.4	82.5	99.1	92.3	8.7		
		3	87.8	81.7	78.8	82.8	4.6		
	500	1	99.2	100	98.1	99.1	1.0	100	1.7
		2	97.9	101.2	98.9	99.3	1.7		
		3	101.1	103.1	100.7	101.6	1.3		
LOD (ng/mL): 9<br>LOQ (ng/mL): 28	1000	1	100.2	100	100.4	100.2	0.2	100.9	2.1
		2	100.6	99.9	100.3	100.3	0.4		
		3	99.9	106.4	100.5	102.3	3.6		

CV, coefficient of variance; LOD, limit of detection; LOQ, limit of quantitation; QC, quality control.
